# Mutations in CREBBP and EP300 HAT and Bromo Domains Drive Hypermutation and Predict Survival in GI Cancers Treated with Immunotherapy

**DOI:** 10.3390/biomedicines13112592

**Published:** 2025-10-23

**Authors:** Mariia Gusakova, Fedor Sharko, Aleksandra Mamchur, Eugenia Boulygina, Anastasia Mochalova, Artem Bullikh, Maxim Patrushev

**Affiliations:** 1 National Research Center “Kurchatov Institute”, 123182 Moscow, Russia; 2 Joint Stock Company “Medsi Group”, 143442 Moscow, Russia; 3 Faculty of Biology, Lomonosov Moscow State University, 119991 Moscow, Russia

**Keywords:** esophagogastric adenocarcinomas, CREBBP, EP300, genomic instability, co-mutations, immunotherapy biomarkers

## Abstract

**Background:** The role of CREBBP and EP300 mutations in hypermutation and immunotherapy response in gastroesophageal adenocarcinomas is poorly defined and needs further investigation. **Methods:** We conducted an in silico analysis of 12 publicly available studies (n = 1871; cBioPortal), stratifying samples by CREBBP/EP300 status to assess associations with TMB-High, MSI, co-mutation patterns, and mutation localization. Clinical validation was performed in an independent pan-cancer cohort treated with ICIs (n = 1610) and a gastric cancer cohort with WES data (n = 55). **Results:** Coding mutations in CREBBP and/or EP300 were significantly associated with TMB-high and MSI-high phenotypes (*p* < 0.001). All studied samples carrying coding mutations in both CREBBP and EP300 exhibited a TMB-high status. PTVs in functional HAT and bromodomain regions were exclusively associated with TMB-high. Incorporating CREBBP and/or EP300 mutation status improved identification of ultra-hypermutated tumors compared with single-gene biomarkers (*p* < 0.001). Clinically, these mutations predicted improved overall survival in the pan-cancer cohort (median OS 34 vs. 17 months; HR = 0.68, 95% CI 0.52–0.87, *p* = 0.0026), as well as in bladder (HR = 0.55, *p* = 0.0337) and gastrointestinal cancer cohorts (HR = 0.31, *p* = 0.0021) treated with ICIs. In the gastric cancer validation cohort, all tumors with PTVs demonstrated a partial response to anti-PD-1 therapy. **Conclusions:** We report CREBBP and EP300 coding mutations as novel potential surrogate biomarkers for hypermutation in gastroesophageal adenocarcinomas and demonstrate their association with favorable immunotherapy outcomes, supporting their potential clinical utility for patient stratification.

## 1. Introduction

Immune checkpoint inhibitors (ICIs) targeting PD-1, PD-L1, and CTLA-4 have profoundly reshaped the therapeutic paradigm in oncology, revolutionizing the management of various malignancies. A key determinant of ICI response is genomic instability, reflected by deficient DNA mismatch repair (dMMR), microsatellite instability (MSI), and an increased tumor mutational burden (TMB). These molecular features are typically associated with high somatic mutation rates, an increased density of tumor-specific neoantigens, and elevated expression of immune checkpoint molecules. They also correlate with an immune-enriched tumor microenvironment containing CD4^+^ and CD8^+^ T cells capable of mounting effective cytotoxic antitumor responses [1,2]. Currently, dMMR/MSI and TMB are established, tumor-agnostic, first-line genomic biomarkers recommended clinically to guide ICIs therapy selection [3].

Despite current advances, some patients remain in a “gray zone”. This is not only due to the lack of novel biomarkers to reliably identify target populations for ICI therapy in clinical practice [4]. It is also because several unresolved issues persist regarding the diagnosis of TMB and MSI status and their role in clinical decision-making [5,6,7]. Intratumoral heterogeneity, tissue-specific characteristics of different tumor types, technical limitations of detection methods, and their inconsistencies remain active areas of research aimed at improving diagnostic accuracy [8,9,10,11,12]. Therefore, strategies that enable the identification of new indications for immunotherapy in real-world clinical practice and enhance the informativeness of NGS analyses of malignancies are currently of paramount importance [13].

Biomarkers associated with chromatin remodeling defects may serve as potential independent predictors of ICIs’ efficacy among patients with gastrointestinal tract tumors. Several studies have reported the significance of inactivation of the SWI/SNF chromatin remodeling complex (Switch/Sucrose Non-Fermentable) in the development of genomic instability and the creation of a favorable environment for ICIs’ efficacy [14]. The role of mutations in chromatin remodeling genes such as ARID1A, ARID1B, ARID2, KMT2C, KMT2D, and others is actively studied, especially in gastrointestinal cancers [15,16,17,18,19]. Loss of function of histone methyltransferases in gastric cancer has been reported in some studies as a key feature of genomically unstable tumors with MSI-high and/or TMB-high status [20,21]. Investigations of chromatin remodeling gene mutations in genomic instability have specifically focused on the role of protein-truncating variants PTVs. [15,22].

The CREBBP and EP300 genes directly interact with proteins of the SWI/SNF complex [23]. They encode the transcriptional coactivators CREB-binding protein (CBP) and E1A-binding protein p300 (EP300), which also possess histone acetyltransferase activity due to specialized domains that mediate chromatin remodeling [24,25,26,27]. Genetic alterations affecting CREBBP and EP300 are well-established oncogenic drivers in hematological malignancies, wherein disruptions of epigenetic modifiers represent critical events underpinning tumorigenesis [28,29,30,31].

The association between CREBBP and EP300 mutations and TMB-High and/or MSI-High status across various solid malignancies has recently become the focus of active investigation [21]. Previous research has largely focused on urothelial carcinoma [25,32,33,34]. In gastrointestinal adenocarcinomas, loss-of-function alterations in CREBBP and EP300 are rare and insufficiently described, with few studies addressing this topic [35,36]. This work examined the relationship between different mutational profiles of CREBBP and EP300 and significant co-mutations in target genes. The predictive value of coding mutations in these genes for response to ICI therapy was also assessed.

## 2. Materials and Methods

### 2.1. Study Cohorts and Data Acquisition

A retrospective in silico analysis was conducted using publicly available data from 12 gastric and esophageal cancer studies [37,38,39,40,41,42,43,44,45,46,47,48] in the cBioPortal online platform (https://www.cbioportal.org/ accessed on 26 August 2025), comprising whole-exome sequencing (WES). It targeted DNA sequencing based on the MSK-IMPACT panel.

### 2.2. TMB and MSI Assessment

For all samples, annotated mutation files, precomputed TMB metrics, and copy number alteration data were provided, whereas MSI status (MSIsensor score) data were available for 788 samples. TMB was defined as the number of nonsynonymous mutations per megabase (Mut/Mb) in the coding region of the genome, with a standard cutoff of 10 Mut/Mb used to classify samples as TMB-High [6]. The cutoff value for the MSIsensor score to define MSI-High status was set at 3.5% (percentage of unstable microsatellites), based on data from the original study and subsequent validation of the tool [49,50,51].

### 2.3. Mutation Classification and Sample Grouping

Samples were stratified into several groups based on the presence of mutations in coding regions and splice sites of the target genes CREBBP and EP300: (1) samples harboring mutations exclusively in the CREBBP gene (n = 76) (hereafter referred to as the CREBBP group); (2) samples with mutations exclusively in the EP300 gene (n = 43) (hereafter the EP300 group); (3) samples with co-occurring mutations in both CREBBP and EP300 (n = 20) (hereafter the CREBBP/EP300 co-mutant group); and 4) samples with wild-type (WT) status for both target genes (hereafter the WT CREBBP/EP300 group) (n = 1732). Detailed information, including patient and tumor sample identifiers selected according to the described groups, corresponding TMB and MSI scores, and histological types, is provided in Supplementary Table S1.

### 2.4. Analysis of Mutation Types and Structural Mapping

The distribution of TMB and MSIsensor scores was analyzed according to mutation types, including missense, nonsense, indels, and splice site mutations. Protein structure models were generated using the AlphaFold3 algorithm [52], based on the amino acid sequences of the proteins under study: CREBBP (UniProt ID Q92793) and EP300 (UniProt ID Q09472). Protein visualization was performed using the PyMOL software package.

### 2.5. Cohorts and Datasets for Prognostic and Predictive Biomarker Evaluation

The analysis of overall survival (OS) and the prognostic significance of CREBBP and EP300 mutational status was performed in an independent clinical pan-cancer cohort from the Memorial Sloan Kettering Cancer Center (MSKCC, n = 1610), in which all patients received immunotherapy with PD-1/PD-L1 and/or CTLA-4 inhibitors [53]. For all participants, the necessary clinical data were retrieved from cBioPortal, including overall survival in months from the first ICI dose, survival status, diagnosis, somatic mutation profiles, and TMB values.

Additionally, WES data from tumor-normal paired samples (n = 55) from the study by S.T. Kim et al. [54] were retrieved, processed, and analyzed. Available clinical data included PD-L1 expression (CPS, IHC), MSI status, EBV status, and response to anti–PD-1 therapy.

### 2.6. Raw Data Processing and Bioinformatics Analysis

The dataset from the study by S.T. Kim et al. [54] was obtained from the European Nucleotide Archive (https://www.ebi.ac.uk/ena accessed on 2 August 2025). Bioinformatic analysis was performed according to Genomic Data Commons (GDC) best practices [55]. Reads were aligned to the GRCh38.d1.vd1 reference genome using BWA [56], followed by processing with SAMtools [57], Picard2 (v3.3.0) (https://broadinstitute.github.io/picard/ accessed on 25 July 2025), and GATK (v4.6.1.0) [58] tools for indel realignment and base quality recalibration. Somatic variant calling was conducted on tumor-normal pairs using Mutect2 (GATK v4.6.1.0), applying a minimum variant allele frequency (VAF) threshold of 10%. Identified variants were filtered against population databases (ExAC, gnomAD) [59] with an allele frequency cutoff of 0.01 to exclude common polymorphisms. Copy number alterations were called with adjustments for tumor purity and ploidy. MSI status was assessed using MSIsensor [49] and MSIsensor-pro (v1.3.0) [60]. TMB was calculated from the filtered Mutect2 output using a dedicated tool (TMB v1.0, https://github.com/bioinfo-pf-curie/TMB accessed on 20 July 2025) and defined as the total number of nonsynonymous mutations per megabase of the coding genome.

### 2.7. Statistical Analysis

To assess differences in the distributions of TMB and MSIsensor scores among groups stratified by mutation status in the target genes, nonparametric statistical methods were employed: the Kruskal–Wallis test followed by Dunn’s post hoc test, as well as the Mann–Whitney U test for pairwise comparisons. Welch’s *t*-test was used to analyze differences in mean values. The strength and direction of associations between mutation status and TMB/MSIsensor levels were evaluated using Spearman’s rank correlation coefficient. Fisher’s exact test was used to analyze relationships between categorical variables. *p*-values were adjusted for multiple tests using the Bonferroni correction or the Benjamini–Hochberg false discovery rate (FDR) method [61], depending on the specific analysis.

OS was analyzed using Kaplan–Meier estimators and Cox proportional hazards models implemented in the Python lifelines package. Kaplan–Meier survival curves were generated for all patients (pan-cancer analysis) and stratified by cancer type. Survival probabilities at 1, 3, and 5 years and median survival times were estimated for each group. Statistical differences between groups were assessed using the log-rank test. For pairwise comparisons between binary groups, Cox proportional hazards models were fitted to estimate hazard ratios (HR) and 95% confidence intervals (CI). Survival curves were plotted with 95% confidence intervals, and detailed statistics—including sample size, median survival, survival probabilities, log-rank *p*-values, and Cox HRs—were tabulated and provided alongside the figures.

All statistical and computational analyses were performed in Jupyter Notebook (v7.3.2) using Python (v3.13.5). Data visualization was conducted with Matplotlib, Seaborn, and Plotly libraries, alongside visualization tools from the pandas and statsmodels packages.

### 2.8. Gene-Enrichment Analysis

KEGG (Kyoto Encyclopedia of Genes and Genomes) pathway analysis [62,63] was carried out using gene set enrichment analysis (GSEA) [64] with a significance threshold of FDR < 0.05. Visualization of enriched paths was performed using the clusterProfiler package.

## 3. Results

### 3.1. Cohort Overview and Study Group Characteristics

Among the analyzed 1871 adenocarcinoma samples from the esophagus, gastroesophageal junction (GEJ), and stomach, 12.83% (n = 240) exhibited TMB ≥ 10 Mut/Mb, 7.54% (n = 141) had TMB ≥ 20 Mut/Mb, 2.24% (n = 42) had TMB ≥ 50 Mut/Mb, 0.37% (n = 7) had TMB ≥ 100 Mut/Mb, and only one sample (0.05%) presented TMB ≥ 200 Mut/Mb. Out of 788 analyzed samples with available MSIsensor scores, 64 samples (8.12%) were classified as MSI-High. The vast majority of these exhibited TMB values exceeding 10 Mut/Mb, except for 13 samples from the group harboring mutations in the CREBBP gene (Supplementary Table S1).

**Figure 1:**
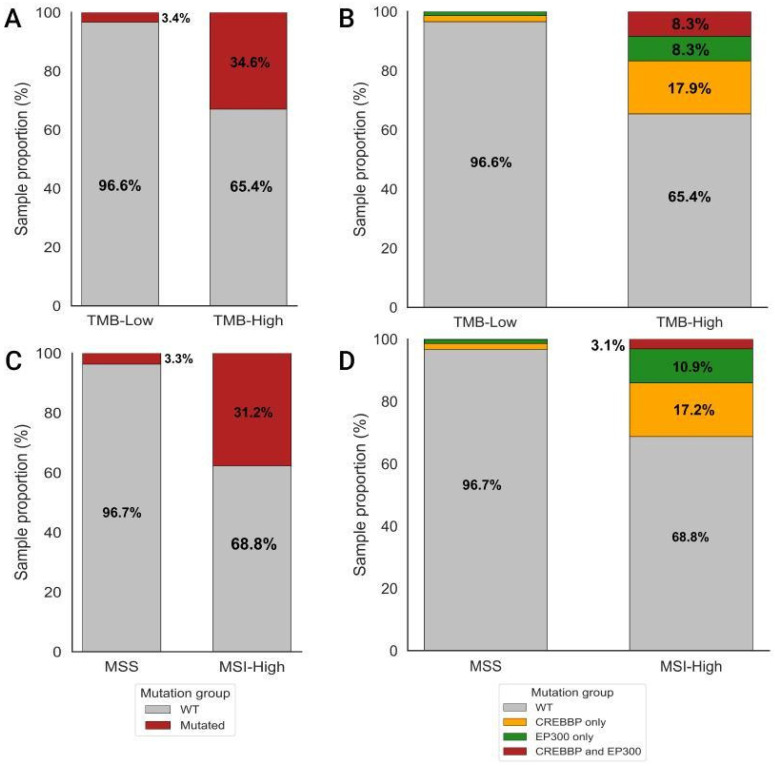
Proportion of samples with mutations in the target genes CREBBP and/or EP300 across TMB and MSI groups. (**A**) Proportion of samples with CREBBP and/or EP300 mutations in the TMB-High (n = 240) and TMB-Low (n = 1631) groups within the overall cohort (n = 1871). (**B**) Distribution of samples harboring CREBBP, EP300, or CREBBP/EP300 co-mutations between the TMB-High and TMB-Low groups (n = 1871). (**C**) Proportion of samples with CREBBP and/or EP300 mutations in the MSI-High (n = 64) and MSS (n = 724) groups within the MSI-assessed cohort (n = 788). (**D**) Distribution of samples with CREBBP, EP300, or CREBBP/EP300 co-mutations between the MSI-High and MSS groups (n = 788).

In the entire cohort, the proportion of samples harboring coding mutations in CREBBP was 5%, and in EP300 2%, with co-mutated samples (CREBBP/EP300) accounting for 1%. Notably, samples with mutations in the target genes were highly represented within the TMB-High and MSI-High groups of the overall cohort (Figure 1).

The distribution of sample counts and their respective proportions across groups defined by mutation status in the coding regions of the target genes CREBBP and EP300, stratified by TMB and MSIsensor score levels, is summarized in Table 1 and illustrated in Supplementary Figure S1.

When analyzing the frequency of mutations across histological tumor types in relation to TMB status, a marked predominance of samples harboring mutations in the target genes was observed within the TMB-High subgroup. Among adenocarcinomas of the GEJ and stomach, the proportion of mutant samples in the TMB-High group was 33% and 39.5%, respectively, compared with 3.9% and 3.3% in the TMB-Low group. In esophageal adenocarcinomas, the proportion of mutant samples in the TMB-High subgroup was 16.2%, approximately two times lower than in gastric and GEJ adenocarcinomas. Notably, among poorly differentiated diffuse-type gastric adenocarcinomas (n = 151), CREBBP and/or EP300 mutations were detected in 47.4% of samples in the TMB-High group. In contrast, in the TMB-Low group, the frequency of CREBBP and/or EP300 mutations did not differ across histological tumor types (Supplementary Figure S2, Supplementary Table S1).

### 3.2. Analysis of Differences in TMB and MSI Values Between CREBBP and/or EP300 Mutant and WT Samples

We identified statistically significant differences in mean TMB values (32.2 vs. 5.953, *p*-value = 1.77 × 10^−14^) and MSIsensor scores (11.139 vs. 1.706, *p*-value = 5.58 × 10^−5^) between tumors harboring mutations in CREBBP and/or EP300 and WT (Welch’s *t*-test). When analyzed separately, CREBBP-mutated tumors retained significantly higher TMB (29.743 vs. 5.953, *p*-value = 1.02 × 10^−7^) and MSI scores (8.657 vs. 1.706, *p*-value = 0.0081) compared to WT samples. Similarly, EP300-mutated tumors exhibited significantly higher mean TMB scores (24.339 vs. 5.953 WT CREBBP\EP300, *p*-value = 10^−4^), and MSI scores (12.372 vs. 1.706, *p*-value = 0.0122). However, interpretation of MSI differences is limited by the small number of mutant samples with known MSI data (CREBBP n = 11; EP300 n = 7).

**Figure 2:**
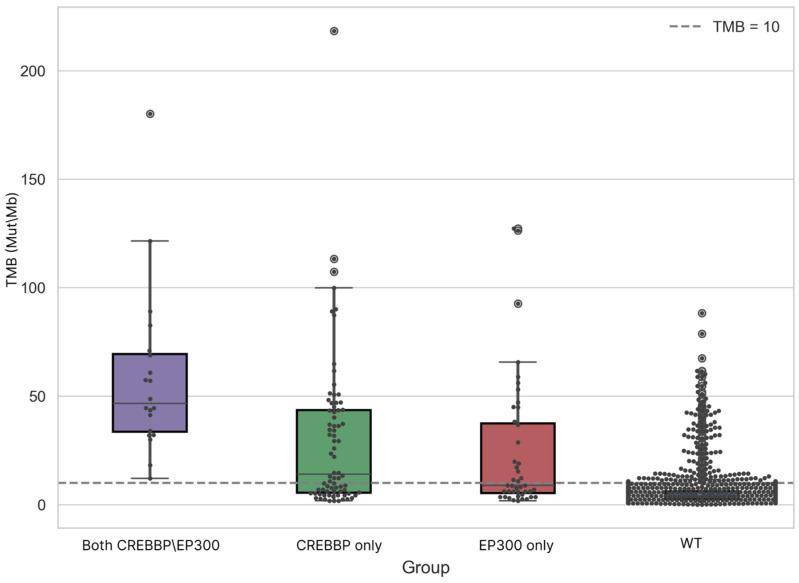
Distribution of TMB and MSI scores between groups of samples containing mutations in target genes and the WT CREBBP/EP300 group.

We performed an analysis of TMB value distributions across the four studied groups, including a statistical assessment of differences between them (Figure 2).

In the group harboring mutations in both genes, the minimum TMB value was 12.11 Mut/Mb (Median [IQR]: 46.55 [33.5–69.40]). Overall, the mean and median TMB values in the groups with mutations in CREBBP and/or EP300 were three times or more higher than those in the WT CREBBP/EP300 group (Median [IQR]: 3.97 [2.53–6.18]) (Table 2).

The differences among the CREBBP, EP300, CREBBP/EP300, and WT CREBBP/EP300 groups (Figure 2) were statistically significant (Kruskal–Wallis test: H = 167.57, *p* = 4.26 × 10^−36^). Pairwise comparisons confirmed highly significant differences between groups, with the exception of tumors containing isolated CREBBP or EP300 mutations (Dunn’s post hoc test, Mann–Whitney U test). When samples were dichotomized by mutational status (Mutated (n = 136) vs. WT (n = 1732)), TMB distributions remained significantly different (Median [IQR]: 18.16 [6.05–45.86] vs. 3.97 [2.53–6.18], Mann–Whitney U test, *p*-value = 2 × 10^−36^) (Supplementary Figure S3).

### 3.3. Correlation Analysis of Genes Carrying Coding Mutations Associated with TMB-High

We analyzed the overall list of genes harboring coding mutations that significantly correlate with TMB-High, using two cutoff thresholds (10 Mut/Mb and 20 Mut/Mb). Spearman’s correlation coefficient was calculated to assess the strength and significance of these associations. This approach allowed us to precisely identify CREBBP and EP300 among the top correlated genes (Table 3, Supplementary Table S2).

EP300 was also among the significantly associated genes, ranking 166th (Spearman’s correlation coefficient with the respective TMB cut-offs: r_1_ = 0.27, *p*-value = 2.2 × 10^−16^; r_2_ = 0.27, *p*-value = 2.2 × 10^−16^).

A separate correlation analysis of coding mutations in CREBBP and/or EP300 with TMB-High also revealed a statistically significant positive correlation r_1_ = 0.39, *p*-value = 4803 × 10^−71^; r_2_ = 0.44, *p*-value = 3.75 × 10^−87^).

### 3.4. Landscape of Genes Co-Mutated with CREBBP and EP300

**Figure 3:**
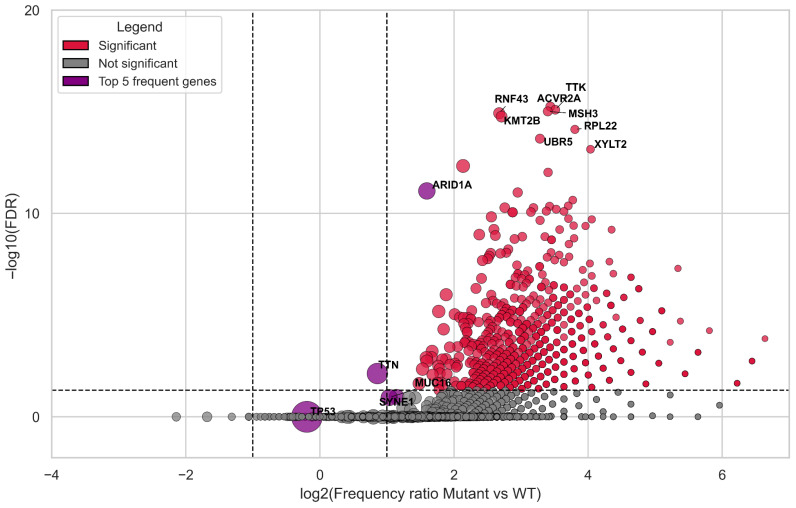
Volcano plot of co-mutations associated with CREBBP/EP300 genes. Purple circles indicate the five most frequently co-mutated genes, while red circles highlight statistically significant associations (FDR-adjusted *p* ≤ 0.05 and |log2 fold change| > 1). The size of each circle is proportional to the prevalence of the corresponding mutation. Statistical comparisons were performed using Fisher’s exact test (two-sided).

We assessed the most frequently co-mutated genes occurring alongside CREBBP and/or EP300 compared to the WT CREBBP/EP300 group. The top 10 most significant co-mutated genes were ACVR2A, MSH3, TTK, RNF43, KMT2B, RPL22, UBR5, XYLT2, KMT2D, TRIO, and ARID1A (*p*-value < 10^−15^). Among the most highly significant genes were all those previously reported in the context of the hypermutator phenotype of gastric cancer, including POLD1 (*p*-value < 10^−11^), POLE, KMT2C, KMT2A (*p*-value < 10^−8^), and MLH1 (*p*-value < 10^−4^) (Figure 3, Supplementary Table S3).

After removing samples with co-mutations in both target genes (CREBBP/EP300) and re-analyzing the remaining sample statistically, the most significant associations remained primarily with MSH3 and KMT2B (*p*-value < 10^−8^). Following in descending order of significance were RNF43, XYLT2, WDTC1, TRIO, ACVR2A, SUPT6H, MBD6, LATS2, POLD1, FAT1, ARID1A, KMT2D, GLI1, BRD4, and MTOR (*p*-value < 10^−5^) (Supplementary Table S4).

**Figure 4:**
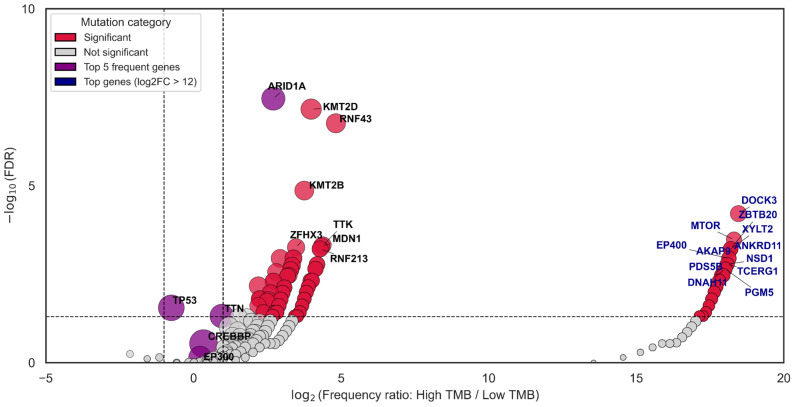
Volcano plot illustrating the results of the co-mutation analysis restricted to tumors harboring mutations in CREBBP and/or EP300, stratified by TMB. Statistically significant genes (FDR < 0.05 and |log_2_FC| > 1) are highlighted in red, whereas genes with the highest overall mutation frequencies are shown in purple, regardless of significance. Genes exhibiting the strongest enrichment (log_2_FC > 12, labeled in blue) were found exclusively in TMB-High tumors within this cohort. A small pseudocount (10^−6^) was added to zero frequencies to enable log-transformation, resulting in their placement at the extreme edges along the x-axis.

Then we focused exclusively on samples carrying mutations in CREBBP and/or EP300 to specifically assess co-mutation patterns between the TMB-High and TMB-Low subgroups within this cohort. This analysis revealed a markedly higher prevalence of co-occurring mutations in ARID1A, KMT2D, and RNF43 in TMB-High samples (*p*-value < 10^−6^) while mutations in MTOR, DOCK3, ZBTB20, ANKRD1, XYLT2, EP400, and others were completely absent in the TMB-Low subgroup (*p*-value < 10^−3^) (Figure 4, Supplementary Table S5).

### 3.5. CREBBP and EP300 as Key Co-Mutants of Genes Associated with TMB-High

**Figure 5:**
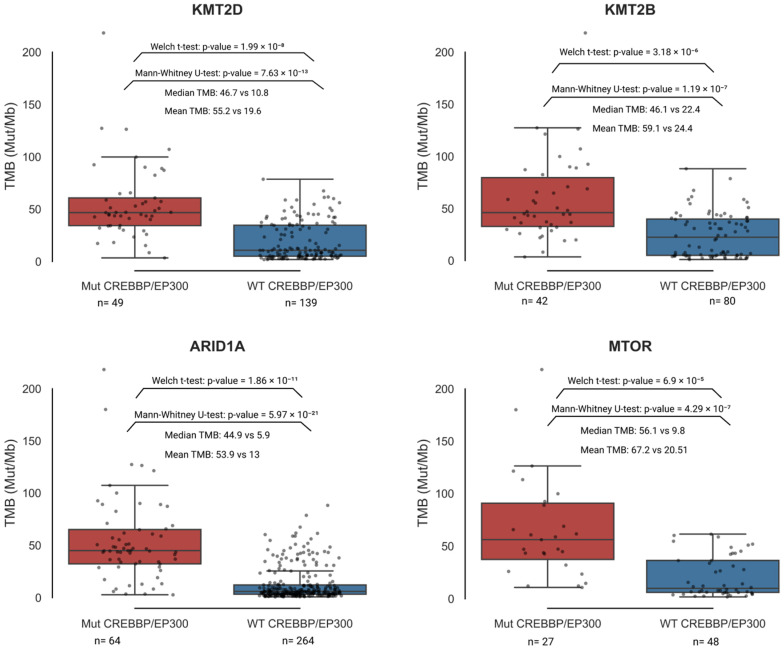
Distribution of TMB values in samples mutated in KMT2D, KMT2B, ARID1A, and MTOR stratified by the presence of co-mutations in CREBBP and/or EP300. In all groups with mutations in ARID1A, KMT2B, KMT2D, and MTOR, the presence of co-mutations in CREBBP and/or EP300 was associated with a significant increase in TMB levels compared to the corresponding WT CREBBP/EP300 group. Detailed statistical results are provided in Supplementary Table S6.

In contrast to the previous analysis, we evaluated the role of CREBBP and EP300 as important components of the co-mutation profile of other genes independently associated with TMB-High (Table 3). Our objective was to evaluate whether including the co-mutated status of CREBBP and/or EP300 would improve TMB-High prediction compared to using the mutational status of individual top-associated genes from Table 3 alone. For each gene listed in Table 3, we performed a separate analysis by selecting samples mutated only in that specific gene and then stratified each subset into CREBBP and/or EP300 mutant versus WT CREBBP/EP300. In all cases, considering CREBBP and/or EP300 as co-mutants with the analyzed gene was statistically significantly associated with the selection of samples exhibiting higher TMB, compared to using the mutation status of the individual gene alone as a predictive biomarker. The mean values in groups harboring mutations in the genes from Table 3 along with co-mutations in CREBBP and/or EP300 were in almost every case more than three times higher. An example of results for KMT2D, KMT2B, ARID1A, and MTOR from the analyzed genes in Table 3 is shown in Figure 5. Complete statistical analysis data are provided in Supplementary Table S6.

**Figure 6:**
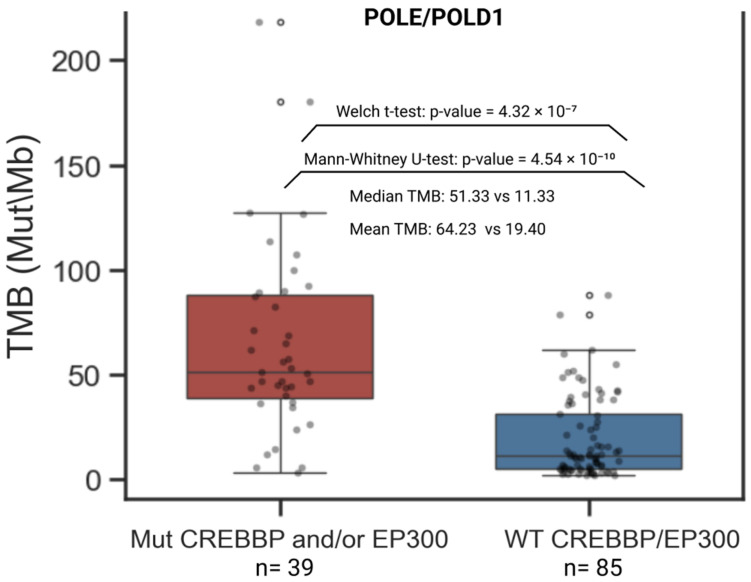
Distribution of samples harboring POLE and/or POLD1 mutations stratified by the presence of mutations in CREBBP and/or EP300 versus the WT CREBBP/EP300 group.

Mutations in POLE and POLD1 are clinically used alongside MSI and TMB to guide immune checkpoint therapy decisions and are incorporated into several clinical guidelines. Therefore, in addition to the analysis performed with the genes listed in Table 3, we assessed differences in TMB between samples mutated in CREBBP and/or EP300 versus the WT CREBBP/EP300 group, restricting the analysis to samples with mutations in POLE and/or POLD1. The result was replicated. Within the CREBBP/EP300 mutant group, samples harboring mutations in DNA polymerase ε and δ genes exhibited significantly higher TMB levels (Mann–Whitney U test: U = 2816.5, *p*-value = 4.54 × 10^−10^; Welch’s *t*-test: t = 5.92, *p*-value = 4.32 × 10^−7^) (Figure 6).

### 3.6. Pathway Enrichment Analysis Among Significant Co-Mutated Genes with CREBBP and EP300

**Figure 7:**
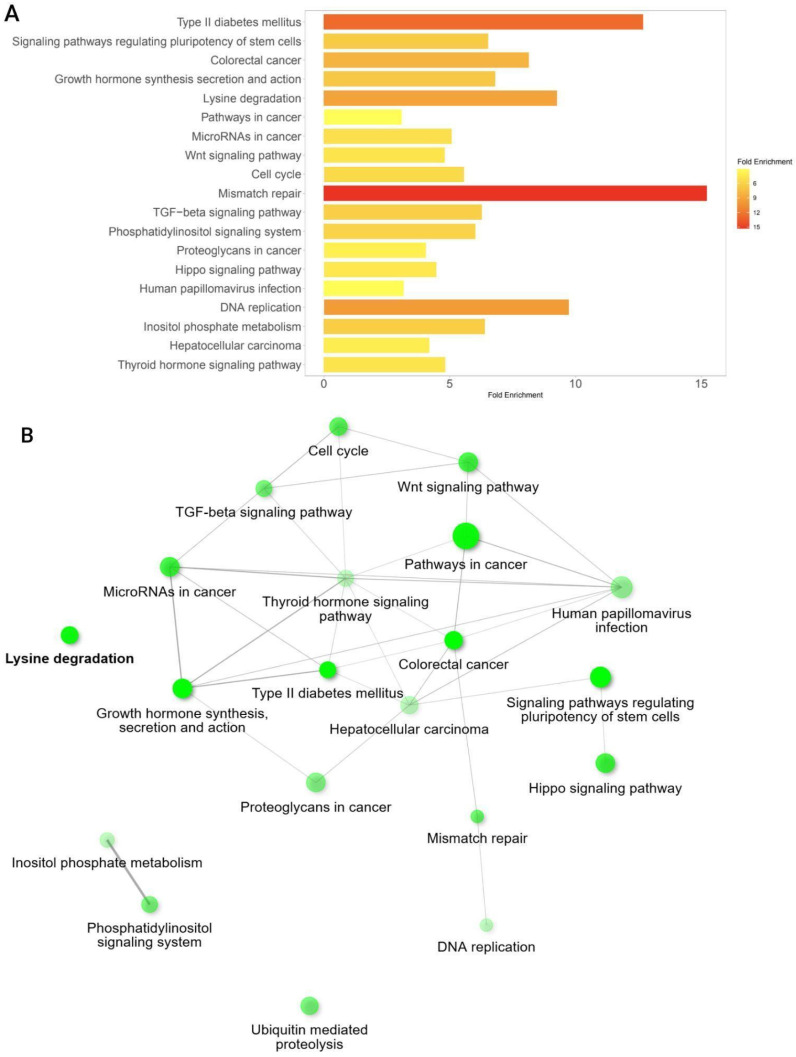
Identification of highly enriched KEGG pathways based on GSEA of genes co-mutated with CREBBP and EP300. (**A**). List of significantly enriched signaling pathways. (**B**). Network representation of interactions among key enriched pathways involving genes co-mutated with CREBBP and EP300.

Using the list of genes co-mutated with CREBBP and/or EP300 (Supplementary Table S3), we evaluated which KEGG signaling pathways were most enriched among the top 200 genes. The results are presented in Figure 7.

Among the most enriched pathways were processes related to the regulation of MMR, Wnt, and Hippo signaling, the cell cycle pathway, molecular pathways associated with type II diabetes mellitus, regulation of stem cell pluripotency, and hormone synthesis and action (Figure 7).

### 3.7. Analysis of Mutation Types and Their Domain Localization Effects in CREBBP and EP300 Genes

**Figure 8:**
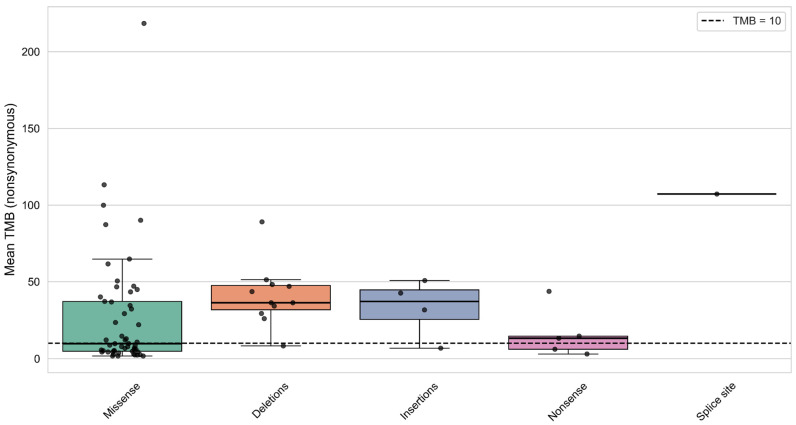
Distribution of samples by TMB level according to mutation type in the CREBBP gene.

In the cohort of samples with CREBBP mutations, differences in TMB levels were observed depending on the mutation type—whether the gene contained only missense mutations or also included nonsense mutations, frameshift deletions, insertions, and splice site mutations (Kruskal–Wallis test: H = 10.312, *p*-value = 0.035). Samples with frameshift mutations formed a subgroup characterized by significantly higher TMB (Figure 8).

The first group includes samples harboring only missense mutations, while the other groups comprise samples with at least one of the specified PTVs. No samples contained multiple PTVs in CREBBP.

**Figure 9:**
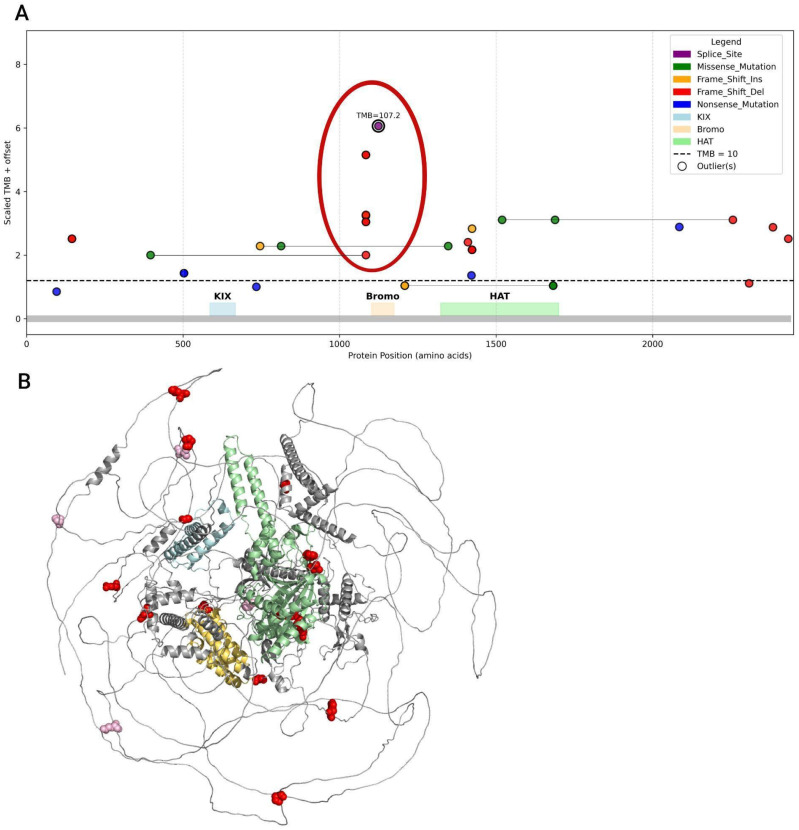
Position of PTVs in the CREBBP gene. (**A**). The plot displays frameshift deletions, insertions, splice site mutations, and mixed variant types. Missense mutations shown on the figure co-occur with PTVs in individual samples; mutations from the same sample are connected by thin gray lines. Samples with only missense mutations are excluded from this analysis. Samples with high TMB are highlighted with a black border and annotated with their TMB values. The height of the circles from the baseline corresponds to the scaled TMB value (TMB multiplied by 0.05 and offset by +0.7 for visualization) in each sample; the mutation with the highest TMB is labeled. Frameshift deletions and splice site mutations clustered at the start of the Bromo domain are outlined with a red contour. Protein domain regions (KIX, Bromo, HAT) are shown as colored rectangles along the X-axis. The black dashed line indicates the TMB cutoff of 10. (**B**). Position of PTV mutations in the CREBBP protein. Protein domains are colored as follows: KIX—light blue, Bromo—yellow, HAT—green. Spheres indicate mutation locations: red for TMB-High samples, pink for TMB-Low samples.

Visualization of mutation distribution by protein position and mutation frequency per site (Figure 9) revealed the highest frequency of frameshift deletions at the start of the bromodomain.

Overall, variants associated with the highest TMB, namely a frameshift deletion starting at amino acid 1084 and a splice site mutation at position 1124, are localized to the bromodomain or its adjacent residues. The greatest number of unique PTVs localized to the HAT domain, which is explained by its larger size relative to other functional domains. Samples from the studied cohort carrying PTV mutations in CBP functional domains exhibited a TMB-High status. The distribution of samples with missense variants in the CREBBP gene is shown in Supplementary Figure S4.

**Figure 10:**
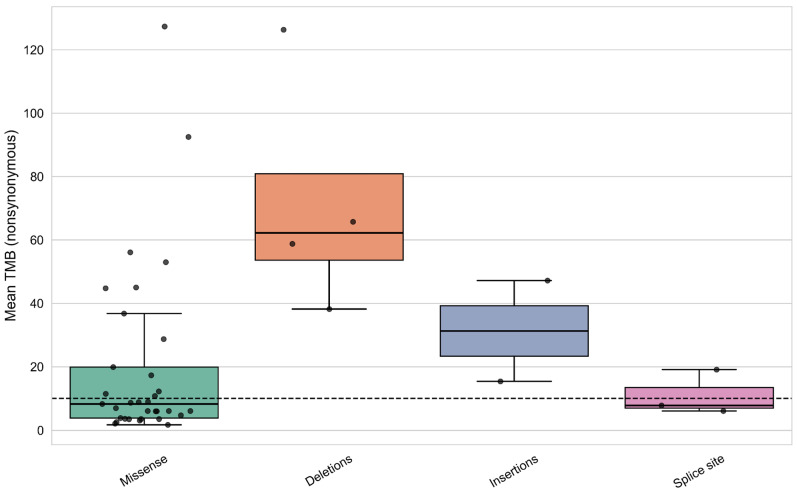
Distribution of samples by TMB level according to mutation types in the EP300 gene.

A similar analysis was performed for the EP300 gene, which showed a comparable trend for frameshift mutations (Figure 10).

**Figure 11:**
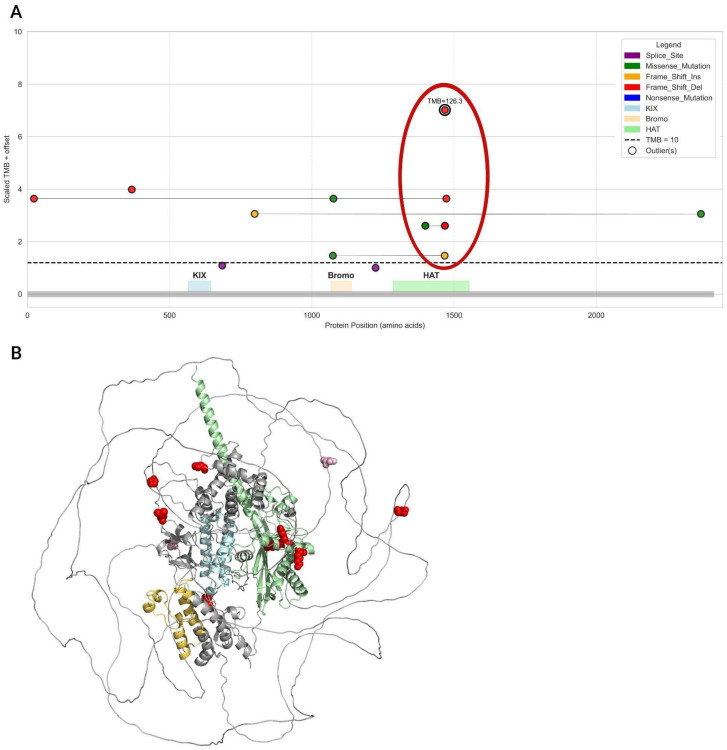
Position of PTVs in the EP300 gene. (**A**). The plot shows frameshift deletions, insertions, splice site mutations, and mixed variant types (samples with only missense mutations are excluded). The color of the points corresponds to the mutation type. The black dashed line marks the TMB cutoff at 10. Samples with high TMB are highlighted with a black border and annotated with their TMB values. Protein domain regions (KIX, Bromo, HAT) are shown as colored rectangles along the X-axis. Frameshift mutations clustered at the HAT domain are outlined with a red contour. (**B**). Position of PTV mutations in the EP300 protein. Protein domains are colored as follows: KIX—light blue, Bromo—yellow, HAT—green. Spheres indicate mutation locations: red for TMB-High samples, pink for TMB-Low samples.

When visualizing the positions of mutations along the protein sequence, it is evident that the vast majority of samples with PTV mutations (except for two) have a TMB-High status. In contrast, samples with only missense mutations fall below the cutoff line (TMB-Low) (Figure 11).

In this case, mutations associated with the highest TMB values were located in the HAT domain. The highest TMB values corresponded to samples with a frameshift deletion starting at amino acid 1468, which was also the most frequently observed mutation. Similar to CREBBP, all PTV variants in EP300 located within functional protein domains were found in samples classified as TMB-High. The distribution of samples containing only missense variants in EP300 is shown in Supplementary Figure S4.

Most variants (both missense and PTV) were located outside of the functional domains. The structures of the studied proteins are not fully resolved and contain extensive intrinsically disordered regions (Figure 9B and Figure 11B, Supplementary Figure S5A,B), which complicates the interpretation of the functional significance of the investigated mutations.

### 3.8. Prognostic and Predictive Significance of CREBBP and EP300 Mutational Status

**Figure 12:**
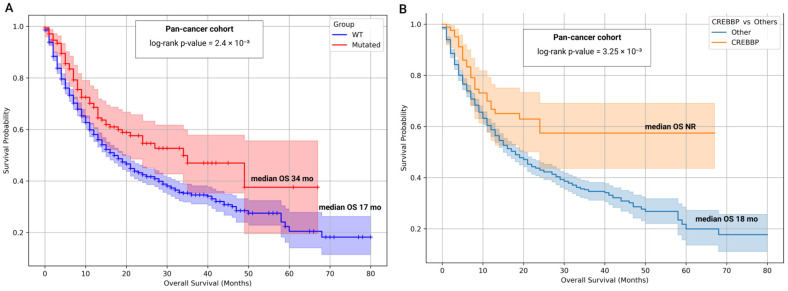
Pan-Cancer Kaplan–Meier Survival Curves: WT vs. Mutated (CREBBP and/or EP300). Survival probability over time (in months) is shown for each group. NR—median OS not reached within the available follow-up period, indicating that more than half of patients were still alive at the last observation. (**A**). Kaplan–Meier survival curves comparing Mutated (CREBBP and/or EP300) versus WT samples in the pan-cancer cohort. Censored events are marked with crosses (+) on the curves. (**B**). Independent analysis of the CREBBP group (n = 84) compared with all other tumors (n = 1526).

We first interrogated the clinicogenomic dataset of the MSKCC pan-cancer cohort (n = 1610) [53]. Tumors harboring mutations in either gene were consolidated into a single Mutated group (n = 172); others are WT CREBBP/EP300 (n = 1438). We then examined their association with overall survival in patients with ICIs (Figure 12).

In the pan-cancer cohort, patients with mutations in the target genes exhibited a median overall survival of 34 months, twice that observed in the WT CREBBP/EP300 group (17 months; log-rank *p*-value = 2.4 × 10^−3^). Cox proportional hazards modeling indicated that the presence of mutation was associated with a reduced risk of death (HR = 0.68; 95% CI = 0.52–0.87; Cox *p*-value = 2.6 × 10^−3^). Time-specific survival estimates further highlighted this trend: at 1, 3, and 5 years, survival probabilities were 58%, 35%, and 21% for WT CREBBP/EP300, versus 69%, 47%, and 38% for Mutated, respectively. Collectively, these findings support an association between mutations and prolonged survival in the pan-cancer cohort.

The following survival analyses focused on subgroups defined by the mutational status of CREBBP and EP300: 84 patients in the CREBBP group, 69 in the EP300 group, and 19 in the CREBBP/EP300 co-mutant group. (Supplementary Figure S6). The CREBBP group (n = 84) did not reach a median OS within the follow-up period. Survival was approximately twice as long as in WT, with consistently higher 1-, 3-, and 5-year survival rates (1y = 67%, 3y = 57%, 5y = 57%). This represented the only statistically significant difference versus WT (log-rank *p*-value = 2.8 × 10^−3^). The EP300 group (n = 69) had a median OS of 21.0 months (1y = 66%, 3y = 35%, 5y = 0%) with no significant difference versus WT (log-rank *p*-value = 0.48). Patients with co-occurring CREBBP and EP300 mutations (n = 19) had a median OS of 34.0 months (1y = 88%, 3–5y = 27%), with a non-significant trend versus WT (log-rank *p*-value = 0.13). Cox regression confirmed the favorable association of the CREBBP group (HR = 0.57; 95% CI: 0.39–0.83; Cox *p*-value = 2.8 × 10^−3^). Neither the EP300 group (HR = 0.88; *p*-value = 0.48) nor the CREBBP/EP300 co-mutant group (HR = 0.54; Cox *p*-value = 0.14) reached statistical significance.

Finally, the independent prognostic impact of CREBBP mutations was further evaluated by comparing the CREBBP group (n = 84) against all other patients in the pan-cancer cohort (WT CREBBP) (Figure 12B). As described above, the CREBBP group did not reach a median OS during follow-up, with 1-, 3-, and 5-year survival rates of 67%, 57%, and 57%, respectively. In contrast, all other patients with WT CREBBP (n = 1526) had a median OS of 18.0 months (1-, 3-, 5-year SR: 59%, 35%, 20%). CREBBP mutations were associated with a significantly reduced risk of death (log-rank *p*-value = 3.25 × 10^−3^; HR = 0.57; 95% CI: 0.39–0.84; Cox *p*-value = 3.84 × 10^−3^), further confirming their favorable prognostic impact on OS in the context of ICIs therapy.

We next repeated the analysis within individual cancer types that had a sufficient number of samples for statistical evaluation. Mutational status in CREBBP and/or EP300 was associated with improved OS in selected gastrointestinal tumors and bladder cancer. In bladder and colorectal cancers, mutated tumors consistently exhibited longer OS than WT CREBBP/EP300 cases, with median OS not reached within the follow-up period in the mutated groups. A similar trend was observed in esophagogastric cancers, although the small number of mutated cases limited statistical significance. Combining esophagogastric and colorectal tumors into a single gastrointestinal cancer group revealed a highly significant association with improved OS. Other tumor types in the cohort showed no significant differences. The overall rarity of CREBBP and EP300 mutations, however, restricts interpretation for individual cancer types. A summary of the survival analysis by cancer type is provided in Table 4 and Supplementary Figure S7.

We also included an additional gastric cancer cohort with available clinical data on response to immunotherapy, PD-L1 expression, and EBV status [54]. Complete clinical information and raw sequencing data were available for only 55 cases, which were analyzed using our bioinformatic pipeline. The number of cases harboring mutations in the target genes was extremely low (n = 6); therefore, only descriptive analyses were performed without formal statistical testing. All of these six studied cases were TMB-High. No mutated samples were EBV-positive. Among them, the three cases with PTV mutations were ultra-hypermutated (TMB > 50 Mut/Mb) and exhibited significant clinical responses to immunotherapy, with PR. Two of these cases with PTVs were co-mutant for CREBBP and EP300, displaying TMB values above 200 Mut/Mb, MSI-High status, and PD-L1 CPS scores of 50 and 100. Another case harbored only missense mutations in CREBBP and EP300, with TMB = 10.3 Mut/Mb and a clinical outcome of stable disease (SD), PD-L1 CPS = 2. Two additional cases with only CREBBP missense mutations corresponded to unfavorable outcomes under ICIs, showing progressive disease (PD). None of the missense mutations in these cases localized to the HAT or Bromo domains. Detailed results are presented in Supplementary Table S7.

## 4. Discussion

As a result of a detailed in silico systematic analysis, our study demonstrates a strong association between coding mutations in the CREBBP and EP300 genes and genomic instability in upper GI adenocarcinomas. Subgroups with high TMB and MSI scores are significantly enriched for mutant samples in CREBBP and/or EP300, especially in GEJ and gastric adenocarcinomas (>30%) and esophageal adenocarcinomas (16%), compared to TMB-Low and MSS groups (3–4%) (Figure 1). This is also true for poorly differentiated diffuse-type gastric cancer (47.4% in TMB-High vs. 3% in TMB-Low) (Supplementary Figure S2, Supplementary Table S1).

Statistical tests confirmed a highly significant difference between the mean values of TMB and MSIsensor scores when comparing samples carrying mutations in the target genes versus WT CREBBP/EP300samples. It held true in the combined analysis of all samples carrying mutations in CREBBP and/or EP300 and in the isolated analysis for each gene individually. Correlation tests confirmed an independent association of CREBBP and EP300 with the TMB-High status, with CREBBP ranking among the top 20 most frequently mutated genes within the TMB-High subgroup of our cohort (Table 3, Supplementary Table S2). A key novel finding of our study was the discovery of a strong association between co-mutations in CREBBP and EP300 and the TMB-High status in the studied combined cohort. CBP and p300 are homologs that partially compensate for each other’s functions. Dual inactivation of these proteins in diffuse large B-cell lymphoma cell models has been shown to result in synthetic lethality [65]. Recent studies have demonstrated that the lysine acetyltransferase activity of CBP and p300 plays an essential role not only in chromatin remodeling but also in orchestrating the activation of key non-histone proteins required for multiple DNA repair pathways, including base excision repair (BER), nucleotide excision repair (NER), non-homologous end joining (NHEJ), and double-strand break repair (DSBR) [66,67]. CREBBP is also crucial for proper homologous recombination repair, acting through direct lactylation of MRN complex proteins [68]. Inactivation of CREBBP and EP300 is considered a therapeutic strategy to achieve tumor radiosensitization due to impaired homologous recombination repair [69]. It is likely that dual inactivation of CREBBP and EP300 in tumors is critical and non-compensable, leading to high hypermutation in cancer genomes. A limitation of this analysis, despite the expanded cohort of nearly 2000 samples, is the rarity of such co-mutations. This should be carefully considered both in the context of biomarker research and in the development of therapeutic agents.

The analysis identified a set of genes, including ACVR2A, MSH3, TTK, RNF43, KMT2B, RPL22, UBR5, XYLT2, KMT2D, TRIO, and ARID1A (Figure 3, Supplementary Table S3), which co-mutate with the target genes of our study. These genes are also among those most strongly associated with the TMB-High status in our analysis (Table 3) and have been described by various authors in the context of genomic instability in solid tumors [70,71]. This also applies to the list of co-mutated genes obtained after removing samples harboring mutations in both CREBBP and EP300 from the cohort (Supplementary Table S4), as well as to the separate analysis of samples mutated only in CREBBP (Supplementary Table S5). Notably, MLH1, MSH2, PMS2, and MSH6 were also identified as significant co-mutants. A similar observation was previously reported in a pan-cancer analysis highlighting EP300 as a marker of genomic instability [21]. Collectively, these findings emphasize that mutations in CREBBP and EP300 represent a critical pattern of the hypermutator phenotype in GI adenocarcinomas.

An enrichment analysis of signaling pathways involving co-mutated genes was also performed. It showed that these genes are predominantly associated with the MMR pathway, and at the same time with regulation of pluripotent stem cells, Wnt, TGF-β, and Hippo signaling pathways—pathways that are typically characteristic of poorly differentiated, aggressive, immune-cold tumors with dysregulation of MHC I and II and resistance to therapy (Figure 7) [72,73,74]. In lymphomas, inactivating mutations in CREBBP and EP300 facilitate effective immune evasion through impaired neoantigen presentation, including in combination with other chromatin remodeling factors [28,75,76]. At the same time, in silico analyses of retrospective data for various solid tumors have shown that mutations in CREBBP and EP300 are associated with immune-inflamed tumors characterized by high neoantigen load, high PD-L1 expression, and a tumor microenvironment rich in T and NK cells, identifying them as potential biomarkers for immunotherapy [21,32,77]. The immunological characteristics of hypermutated GI cancers with altered CREBBP and EP300 genes, the efficacy of immunotherapy, and patients’ outcomes should be thoroughly explored in future studies.

Moreover, we demonstrated for the first time that the co-occurrence of CREBBP and/or EP300 mutations with other genes associated with TMB-High enhances the stratification of TMB-High samples. This is more effective than using individual genes as hypermutation biomarkers. (Figure 5 and Figure 6, Supplementary Table S6). For example, within the MTOR-mutant subgroup, samples harboring concurrent mutations in CREBBP and/or EP300 were exclusively TMB-High (Supplementary Table S6). Samples harboring mutations in the POLE and POLD1 genes were analyzed separately (Figure 6), as these mutations are established independent clinical biomarkers of genomic instability and immunotherapy efficacy across multiple solid tumors, including gastrointestinal cancers. Both POLE and POLD1 mutations are incorporated into clinical guidelines for the management of endometrial and colorectal cancers [78,79,80]. The mutational status of CREBBP and EP300 was significantly associated with higher TMB levels among samples already mutated in POLE/POLD1. These findings can immediately aid the interpretation of NGS tests in clinical practice and help guide the extent of further patient investigations, especially since TMB and MSI status are not always reported.

Next, we leveraged the available data to explore in depth the relationship between mutation types and their domain positions in CREBBP and EP300 with protein function and genomic instability. Our analysis approached this from several angles: (1) assessing the impact of mutation type on sample TMB levels; (2) correlating mutations at specific protein model positions with their mutation type and corresponding TMB status in samples. This approach revealed that nearly all frameshift mutations were associated with TMB-High samples. Moreover, we found that PTVs predominantly localized within highly conserved functional domains of CREBBP and EP300—namely, Bromo and HAT—in all tumors linked to TMB-High status. Further, our analysis pinpointed critical hotspot positions with the highest mutation frequency overall: position 1084, marking the start of the bromodomain in CREBBP, and position 1468 within the HAT domain of EP300 (Figure 9 and Figure 11).

The obtained results align with the current understanding of the functional domains of CBP and p300 proteins. It is likely that PTV variants located directly within or near the HAT domain disrupt the integrity of the core region responsible for histone acetylation [28]. However, our study identified variants in both CREBBP and EP300 that are located downstream of the HAT domain, closer to the C-terminus of the protein, which are also associated with TMB-High status in carriers. These include frameshift deletion variants in CREBBP (Figure 9) and missense variants in both EP300 and CREBBP (Supplementary Figure S4). Interpretation of the nature of this association is complicated by the lack of detailed knowledge regarding the structural organization of this protein region. Moreover, the absence of a full-length protein structure hinders in silico investigations of CREBBP and EP300 interactions with other chromatin remodeling components and potential therapeutic agents.

Taken together, our results establish coding mutations in CREBBP and EP300 as functionally and clinically relevant alterations linked to genomic instability and enhanced tumor immunogenicity. Importantly, analysis of independent patient cohorts receiving ICIs underscored their predictive value in the clinical setting. In the pan-cancer cohort [53], the presence of mutations in at least one of these genes was associated with a two-fold improvement in survival compared with WT CREBBP/EP300 tumors (Figure 12). This association was consistently observed in bladder cancer, colorectal cancer, and in the combined gastrointestinal cancer cohorts. Although a similar but not statistically significant trend was noted in the esophagogastric cancer cohort, the limited number of mutated cases likely reduced statistical power (Table 4, Supplementary Figure S7).

In an additional analysis of 55 WES gastric adenocarcinomas from patients treated with anti–PD-1 therapy in the second- or third-line setting [54], only six cases harbored coding mutations in CREBBP and/or EP300. Strikingly, all six exhibited a TMB-high phenotype. Notably, tumors with PTVs in the target genes consistently showed an ultramutated profile and partial responses to immunotherapy (Supplementary Table S7). Furthermore, two cases with co-occurring PTVs in CREBBP and EP300 displayed exceptionally high TMB (>200 Mut/Mb) and MSI-high status. While the tumor-specific frequency of CREBBP and EP300 mutations constrains the analysis in small cohorts, these findings further support their role as biomarkers of immunotherapy response and underscore the need for expanded datasets and prospective validation.

## 5. Conclusions

We propose that tumors harboring inactivating mutations in the histone acetyltransferases CREBBP and EP300 may represent a distinct molecular subset within the broader group of TMB-high and/or MSI-high gastrointestinal cancers. These tumors likely exhibit unique mechanisms of genomic instability and warrant dedicated investigation to better elucidate their biological behavior, clinical characteristics, and potential therapeutic vulnerabilities.

Our in silico analysis provides preliminary evidence that could support a more accurate interpretation of NGS panel and whole-exome sequencing data in esophageal and gastric cancers. Further retrospective and prospective validation is needed to confirm the role of CREBBP and EP300 mutations as predictive biomarkers of ICI response and to explore their utility in guiding personalized immunotherapy strategies.

## 6. Limitations

This study has several limitations. First, MSI sensor scores were available for less than half of the samples, which may affect the robustness of MSI-related findings. Second, the three-dimensional structures of CBP and p300 proteins remain incompletely resolved, limiting the precision of structural analyses and their interpretation in the context of specific mutations. Third, although we included two independent cohorts and demonstrated the predictive effect of the biomarkers, the number of cases with CREBBP/EP300 mutations in esophagogastric cancers was very small, necessitating larger cohorts for more reliable analysis.

## Figures and Tables

**Table 1:** Absolute and relative counts of samples stratified by TMB and MSI thresholds.

Group	Samples Analyzed for TMB	TMB ≥ 10 (n, %)	TMB ≥ 20 (n, %)	TMB ≥ 50 (n, %)	TMB ≥ 100 (n, %)	TMB ≥ 200 (n, %)	Samples Analyzed for MSI	MSI-High (Score ≥ 3.5) (n, %)
**WT**	1732	157 (9.1%)	74 (4.3%)	13 (0.8%)	0	0	744	44 (5.9%)
**CREBBP**	76	43 (56.6%)	36 (47.4%)	13 (17.1%)	3 (3.9%)	1 (1.3%)	25	11 (44.0%)
**EP300**	43	20 (46.5%)	13 (30.2%)	7 (16.3%)	2 (4.7%)	0	16	7 (43.8%)
**CREBBP/EP300**	20	20 (100%)	18 (90.0%)	9 (45.0%)	2 (10.0%)	0	3	2 (66.7%)

**Table 2:** Statistical summary of TMB values for each group.

Group	Median	Mean	Min.	Max.	Q1	Q3	IQR
**CREBBP/EP300**	46.55	58.44	12.11	180.13	33.50	69.40	35.90
**CREBBP**	13.99	29.74	1.73	218.47	5.55	43.59	38.04
**EP300**	8.81	24.34	1.73	127.33	5.30	37.47	32.17
**WT**	3.97	5.95	0.03	88.20	2.53	6.18	3.64

**Table 3:** The top 30 most frequently mutated genes in tumors correlated with TMB-High in the studied cohort.

Gene	Mutated	WT	Correlation, r_1_ (TMB-High, Cut-Off = 10 Mut\Mb)	Correlation, r_2_ (TMB-High, Cut-Off = 20 Mut\Mb)
KMT2D	181	1705	0.51	0.56
ACVR2A	77	1809	0.46	0.55
KMT2B	120	1766	0.44	0.57
MSH3	74	1812	0.44	0.54
RNF43	125	1761	0.42	0.56
ARID1A	332	1554	0.41	0.46
RPL22	56	1830	0.41	0.52
TTK	69	1817	0.41	0.49
DOCK3	77	1809	0.40	0.46
XYLT2	47	1839	0.39	0.51
BRCA2	96	1790	0.37	0.41
ZFHX3	111	1775	0.37	0.45
IRS1	58	1828	0.36	0.48
UBR5	72	1814	0.36	0.45
MTOR	75	1811	0.35	0.39
UPF3A	39	1847	0.35	0.42
MBD6	51	1835	0.35	0.41
PGM5	45	1841	0.35	0.46
CREBBP	92	1794	0.35	0.40
RGS12	58	1828	0.35	0.42
HLA-B	52	1834	0.35	0.41
KMT2C	124	1762	0.35	0.38
PDS5B	46	1840	0.35	0.38
RNF213	71	1815	0.34	0.40
TRIO	53	1833	0.34	0.43
ZBTB20	49	1837	0.34	0.44
PHF2	45	1841	0.34	0.45
POLE	65	1821	0.34	0.39
TCERG1	43	1843	0.33	0.43
LARP4B	43	1843	0.33	0.38

**Table 4:** Survival analysis by cancer type (Mutated vs. WT CREBBP/EP300).

Cancer Type	WT (n)	Mutated (n)	Median OS WT (mo)	Median OS Mut (mo)	Log-Rank *p*-Value	Cox HR	95% CI	Cox *p*-Value
Pan-Cancer	1438	172	17.0	34.0	0.0024	0.68	0.52–0.87	0.0026
Bladder Cancer	166	45	16.0	NR	0.0306	0.55	0.31–0.95	0.0337
Colorectal Cancer	87	22	13.0	NR	0.0048	0.25	0.09–0.71	0.0089
Gastrointestinal Cancers	195	32	13.0	NR	0.0012	0.31	0.14–0.65	0.0021
Esophagogastric Cancer	108	10	15.0	NR	0.2125	0.48	0.15–1.58	0.2291
Cancer of Unknown Primary	73	12	9.0	NR	0.0800	0.30	0.07–1.26	0.1004
Glioma	108	8	13.0	13.0	0.5154	0.72	0.26–1.97	0.5206
Head and Neck Cancer	118	11	11.0	9.0	0.5012	0.74	0.32–1.74	0.4944
Melanoma	276	37	42.0	49.0	0.9580	1.02	0.57–1.81	0.9595
Non-Small Cell Lung Cancer	319	25	11.0	14.0	0.4410	0.81	0.48–1.37	0.4314

Notes: Gastrointestinal cancers—a combined group including esophagogastric and colorectal cancers.

## Data Availability

The processed data used in this study were obtained from the open-access platform cBioPortal for Cancer Genomics (https://www.cbioportal.org 26 August 2025). Key datasets required for reproducing the analyses are provided in the Supplementary Materials. Additional information, including analysis scripts, is available from the corresponding author upon reasonable request.

## Supplementary Materials

The following supporting information can be downloaded at: https://www.mdpi.com/article/10.3390/biomedicines13112592/s1. Supplementary Table S1. Distribution of sample groups with available histological annotations and corresponding TMB and MSI status. Supplementary Table S2. Results of correlation analysis of genes harboring coding mutations significantly associated with TMB-High. Supplementary Table S3. Results of co-mutation analysis identifying genes significantly co-mutated with CREBBP and/or EP300. Supplementary Table S4. Results of co-mutation analysis identifying genes significantly co-mutated with CREBBP and/or EP300 after exclusion of samples harboring simultaneous CREBBP and EP300 mutations. Supplementary Table S5. Co-mutation profiles in TMB-Low versus TMB-High samples harboring CREBBP and/or EP300 mutations. Supplementary Table S6. Impact of CREBBP and EP300 co-mutational status on the selection of TMB-High samples among other top TMB-associated genes. Supplementary Table S7. Results of the independent gastric cancer cohort analysis. Supplementary Figure S1. Proportion of patients across groups stratified by TMB threshold levels. Supplementary Figure S2. Frequency of mutations in the target genes across histological tumor types. Supplementary Figure S3. Distribution of TMB and MSI scores between samples harboring mutations in target genes and WT CREBBP/EP300 samples. Supplementary Figure S4. Positions of missense variants in CREBBP and EP300 genes. This analysis includes only samples harboring missense mutations, with samples containing PTV variants excluded. Supplementary Figure S5. In silico structural mapping of variant positions in EP300 and CREBBP proteins. Supplementary Figure S6. Kaplan–Meier survival analysis of overall survival in the pan-cancer cohort stratified by four mutational groups. Supplementary Figure S7. Kaplan–Meier analysis of overall survival by CREBBP/EP300 mutational status in selected tumors.

## Abbreviations

BER	Base Excision Repair
CI	Confidence Interval
CREBBP	CREB-binding protein
dMMR	Deficient Mismatch Repair
DSBR	Double-Strand Break Repair
EBV	Epstein–Barr Virus
EP300	E1A Binding Protein P300
FDR	False Discovery Rate
GEJ	Gastroesophageal Junction
GSEA	Gene Set Enrichment Analysis
HAT	Histone Acetyltransferase
HR	Hazard Ratio
HRR	Homologous Recombination Repair
ICIs	Immune Checkpoint Inhibitors
IQR	Interquartile Range
KEGG	Kyoto Encyclopedia of Genes and Genomes
MMR	Mismatch Repair
MSI	Microsatellite Instability
NER	Nucleotide Excision Repair
NGS	Next-Generation Sequencing
NHEJ	Non-Homologous End Joining
OR	Odds Ratio
OS	Overall Survival
PD	Progressive Disease
PR	Partial Response
PTV	Protein-Truncating Variant
SD	Stable Disease
SNV	Single Nucleotide Variant
TMB	Tumor Mutational Burden
WES	Whole-Exome Sequencing
WT	Wild-Type
