# Supplementary material for: Mutations in CREBBP and EP300 HAT and Bromo Domains Drive Hypermutation and Predict Survival in GI Cancers Treated with Immunotherapy
Source: Biomedicines. 2025 Oct 23;13(11):2592. doi: 10.3390/biomedicines13112592 (PMC12650065; doi:10.3390/biomedicines13112592)
Supplement: Supplementary file 1 [file biomedicines-13-02592-s001.zip › Supplementary Figures.pdf]

Supplementary Figures

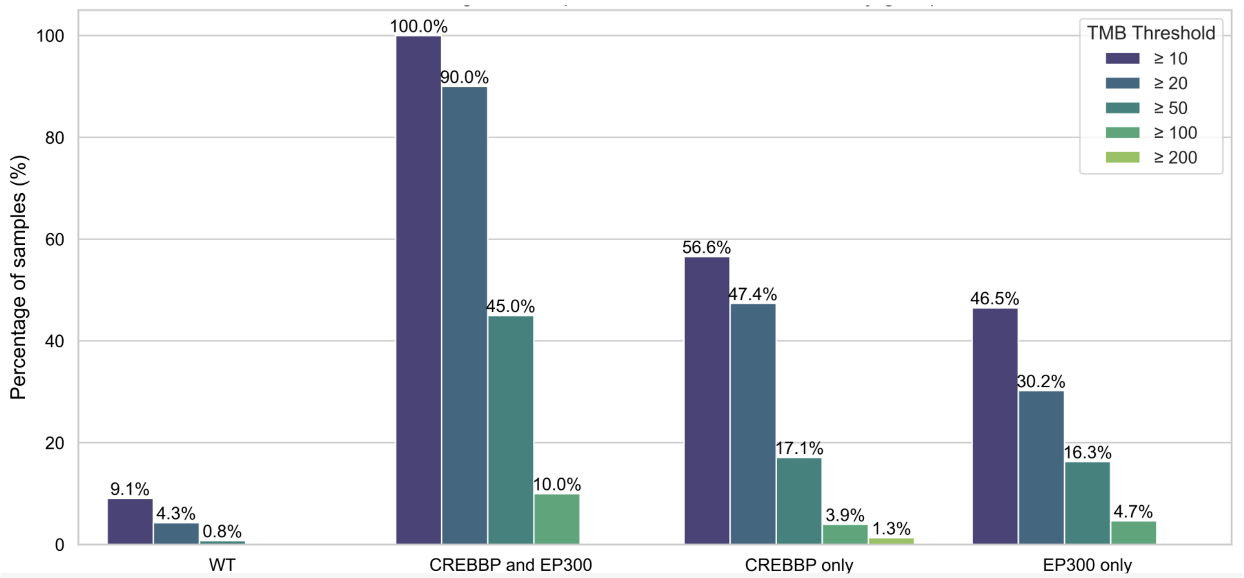

Supplementary Figure S1. Proportion of patients across groups stratified by TMB threshold levels. Approximately half of the samples in the CREBBP and EP300 groups exhibit TMB ≥ 10 Mut/Mb, whereas 100% in the CREBBP/EP300 co-mutant group are classified as TMB-High.

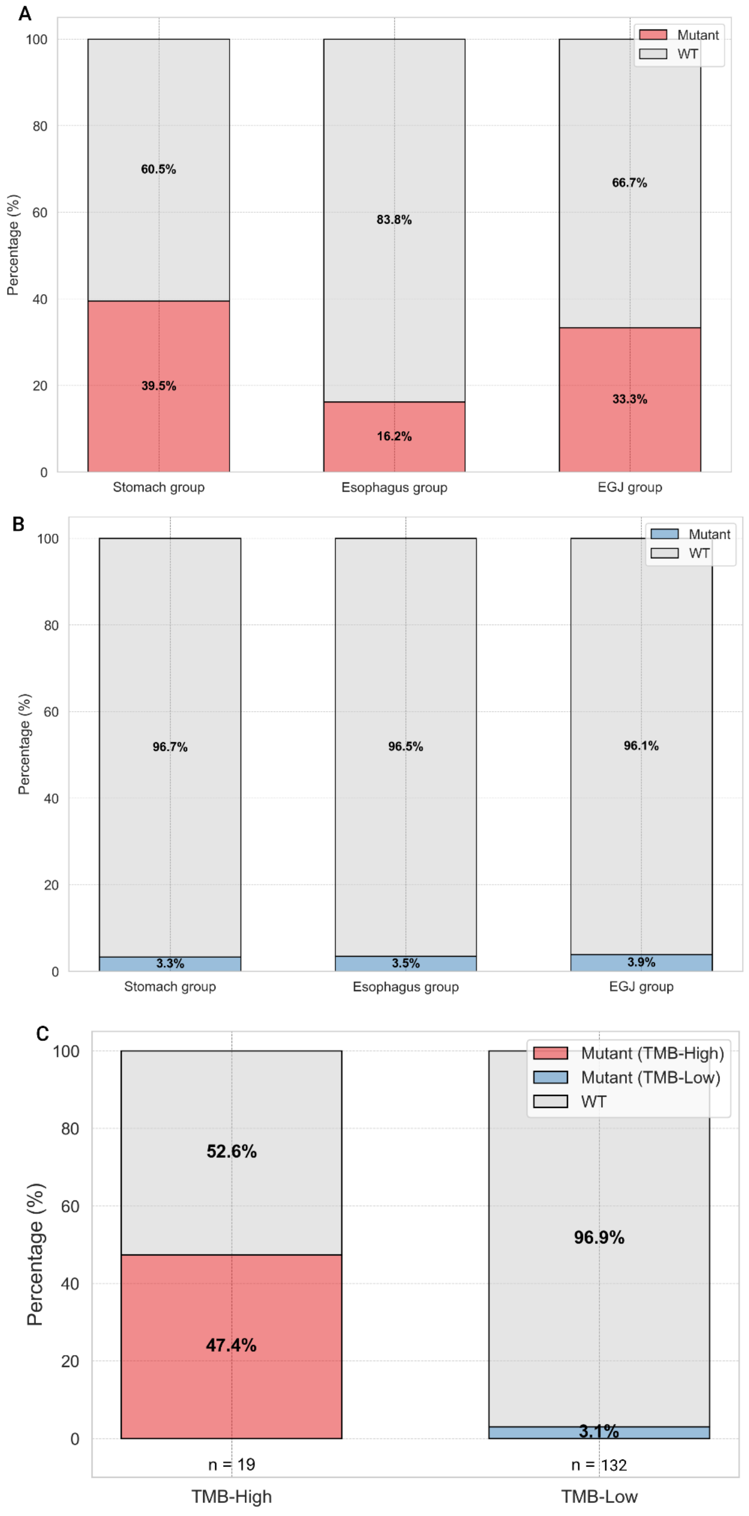

Supplementary Figure S2. Frequency of mutations in the target genes across histological tumor types. **A.** Proportion of CREBBP and/or EP300 mutant and WT samples in the TMB-High subgroup. **B.** Proportion of CREBBP and/or EP300 mutant and WT samples in the TMB-Low subgroup. **C.** Proportion of CREBBP and/or EP300 mutant and WT samples in poorly differentiated diffuse-type gastric cancer across TMB-High and TMB-Low subgroups.

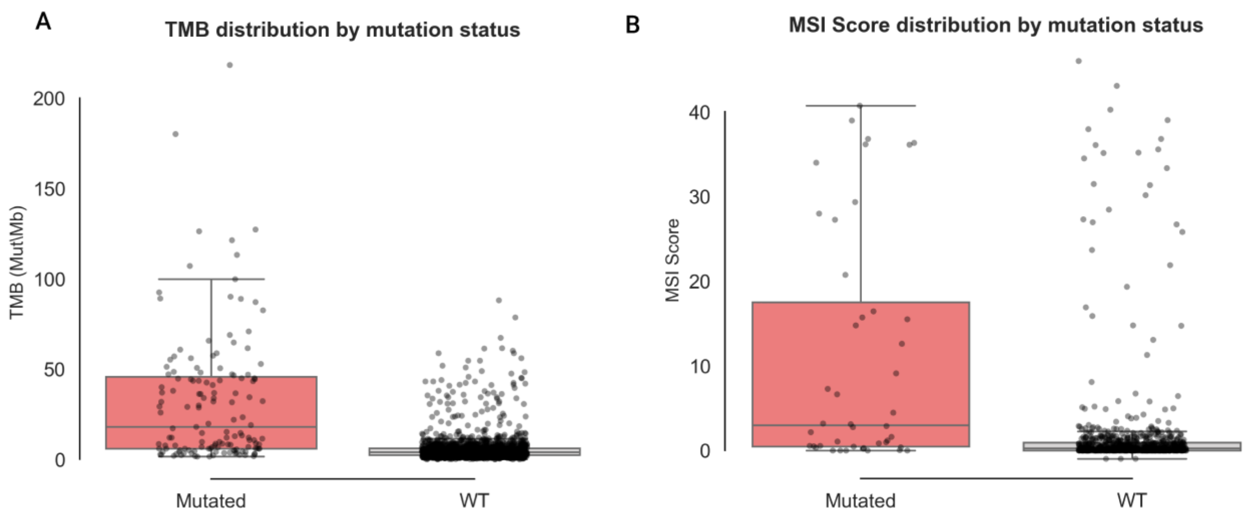

Supplementary Figure S3. Distribution of TMB and MSI scores between samples harboring mutations in target genes and WT CREBBP/EP300 samples. **A.** Distribution of TMB values. **B.** Distribution of MSIsensor scores.

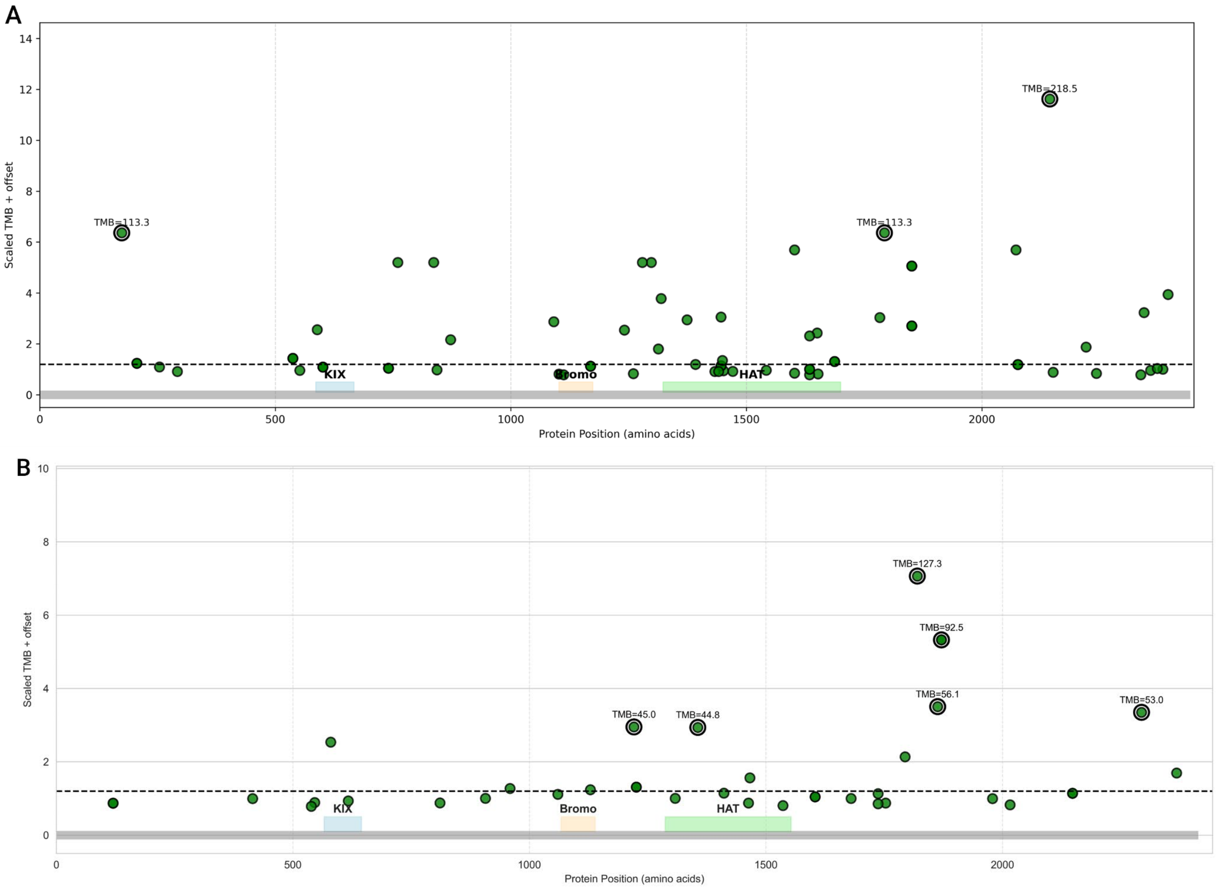

Supplementary Figure S4. Positions of missense variants in CREBBP and EP300 genes. This analysis includes only samples harboring missense mutations, with samples containing PTV variants excluded. The black dashed line indicates the TMB cutoff of 10. Samples with high TMB are outlined in black and annotated with their TMB values. Protein domain regions (KIX, Bromo, HAT) are depicted as colored rectangles along the X-axis. **A.** Positions of missense variants in the CREBBP gene. **B.** Positions of missense variants in EP300 gene.

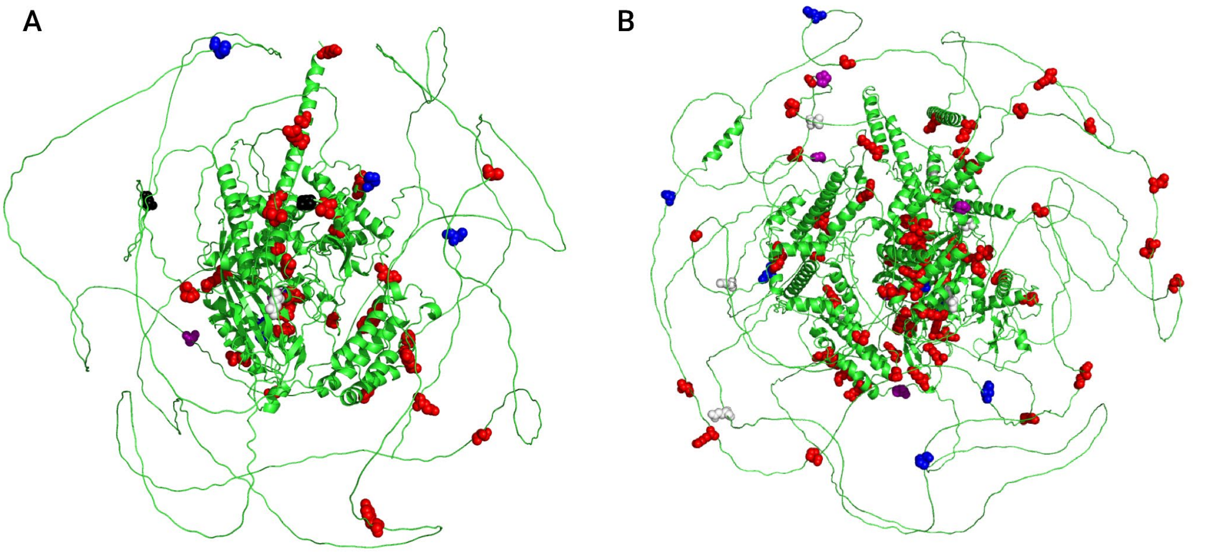

Supplementary Figure S5. *In silico* structural mapping of variant positions in target proteins of study: **A.** EP300; **B.** CREBBP. The color of the points corresponds to the mutation type: red

- missense, blue - frameshift insertions, magenta - frameshift deletions, white - nonsense. black - stopgain.

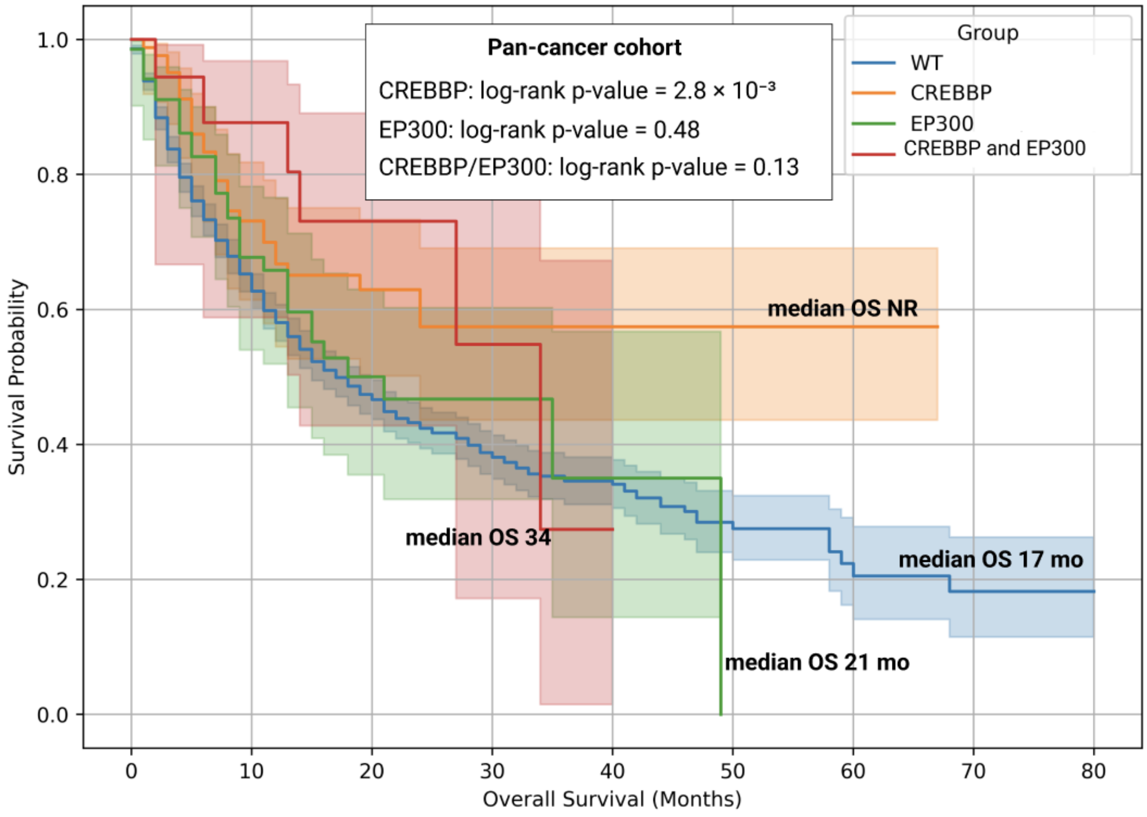

Supplementary Figure S6. Kaplan–Meier survival analysis of overall survival in the pan-cancer cohort stratified by four mutational groups: WT CREBBP/EP300 (n = 1438), CREBBP group (n = 84), EP300 group (n = 69), and CREBBP/EP300 co-mutant group (n = 19).

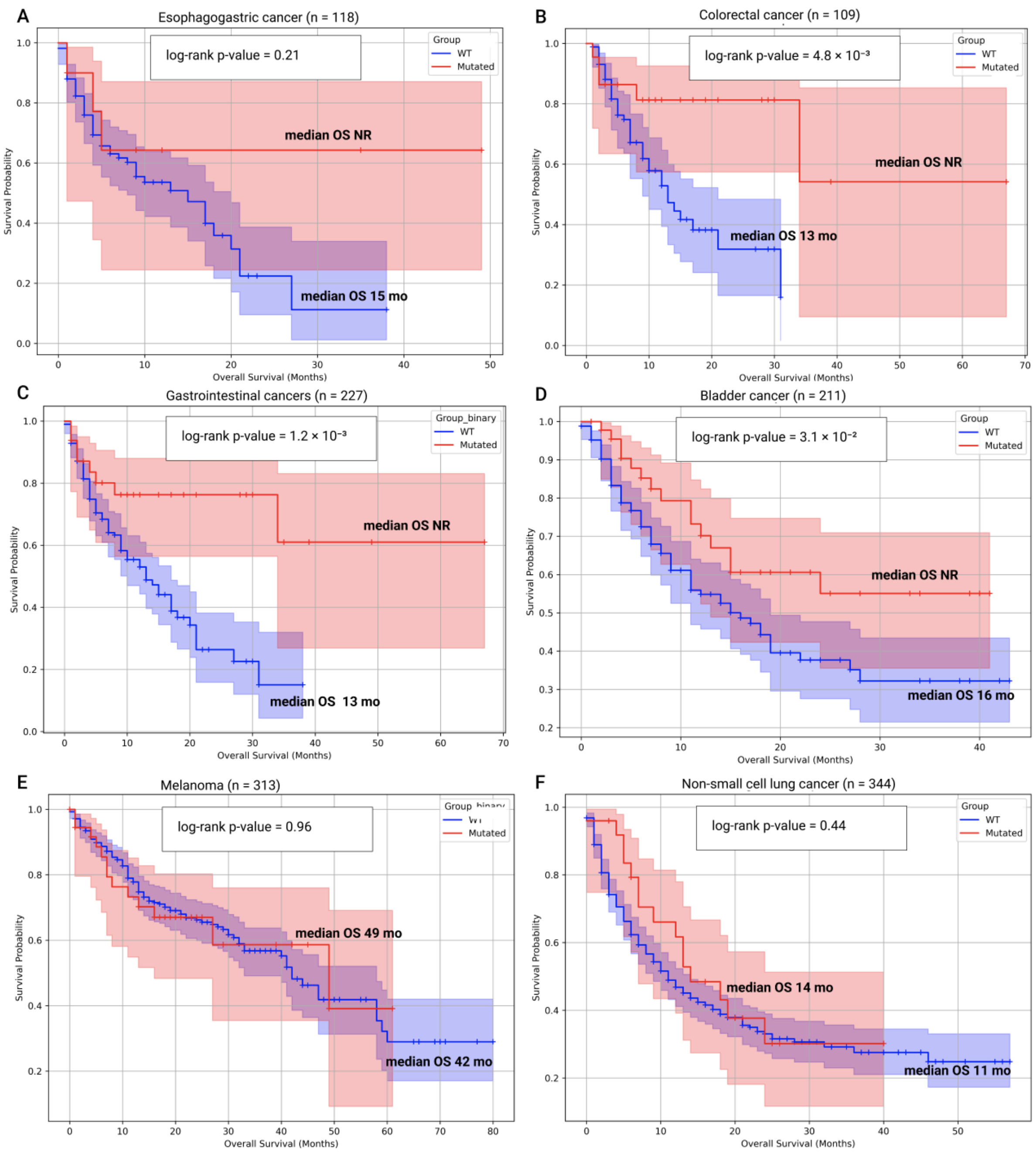

Supplementary Figure S7. Kaplan–Meier analysis of overall survival by CREBBP/EP300 mutational status in selected tumors. **A.** Esophagogastric cancers. **B.** Colorectal cancer. **C.** Gastrointestinal cancers. **D.** Bladder cancer. **E.** Melanoma. **F.** Non-small cell lung cancer.
